# Protocol for the ProFHER (PROximal Fracture of the Humerus: Evaluation by Randomisation) trial: a pragmatic multi-centre randomised controlled trial of surgical versus non-surgical treatment for proximal fracture of the humerus in adults

**DOI:** 10.1186/1471-2474-10-140

**Published:** 2009-11-16

**Authors:** Helen Handoll, Stephen Brealey, Amar Rangan, David Torgerson, Laura Dennis, Alison Armstrong, Ling-Hsiang Chuang, Ben Cross, Jo Dumville, Sarah Gardner, Lorna Goodchild, Sharon Hamilton, Catherine Hewitt, Rajan Madhok, Nicola Maffulli, Lucy Micklewright, Valerie Wadsworth, Angus Wallace, John Williams, Gill Worthy

**Affiliations:** 1 Centre for Rehabilitation Sciences, Health Sciences and Social Care Institute, Teesside University, Middlesbrough, Tees Valley, TS1 3BA, UK; 2 Department of Health Sciences, University of York, Heslington, York, YO10 5DD, UK; 3 Department of Trauma and Orthopaedics, South Tees Hospitals NHS Trust, The James Cook University Hospital, Marton Road, Middlesbrough, TS4 3BW, UK; 4 Department of Trauma and Orthopaedics, University Hospitals of Leicester, Leicester General Hospital, Gwendolen Road, Leicester, LE5 4PW, UK; 5 Department of Rehabilitation, James Cook University Hospital, Marton Road, Middlesbrough, TS4 3BW, UK; 6 NHS Manchester, Southmoor House, Wythenshawe, Manchester, M23 9LH, UK; 7 Centre for Sports and Exercise Medicine, Queen Mary University of London, Barts and The London School of Medicine and Dentistry, Mile End Hospital, 275 Bancroft Road, London, E1 4DG, UK; 8 Division of Orthopaedic & Accident Surgery, University of Nottingham, West Block, C Floor, Queen's Medical Centre Campus, Nottingham, NG7 2UH, UK; 9 Upper Limb Unit, Freeman Hospital, High Heaton, Newcastle upon Tyne, NE7 7DN, UK; 10 University of Leeds, Clinical Trials Research House, 71-75 Clarendon Road, Leeds, LS2 9PH, UK

## Abstract

**Background:**

Proximal humeral fractures, which occur mainly in older adults, account for approximately 4 to 5% of all fractures. Approximately 40% of these fractures are displaced fractures involving the surgical neck. Management of this group of fractures is often challenging and the outcome is frequently unsatisfactory. In particular it is not clear whether surgery gives better outcomes than non-surgical management. Currently there is much variation in the use of surgery and a lack of good quality evidence to inform this decision.

**Methods/Design:**

We aim to undertake a pragmatic UK-based multi-centre randomised controlled trial evaluating the effectiveness and cost-effectiveness of surgical versus standard non-surgical treatment for adults with an acute closed displaced fracture of the proximal humerus with involvement of the surgical neck. The choice of surgical intervention is left to the surgeon, who must use techniques that they are fully experienced with. This will avoid 'learning curve' problems. We will promote good standards of non-surgical care, similarly insisting on care-provider competence, and emphasize the need for comparable provision of rehabilitation for both groups of patients.

We aim to recruit 250 patients from a minimum of 18 NHS trauma centres throughout the UK. These patients will be followed-up for 2 years. The primary outcome is the Oxford Shoulder Score, which will be collected via questionnaires completed by the trial participants at 6, 12 and 24 months. This is a 12-item condition-specific questionnaire providing a total score based on the person's subjective assessment of pain and activities of daily living impairment. We will also collect data for other outcomes, including general health measures and complications, and for an economic evaluation. Additionally, we plan a systematic collection of reasons for non-inclusion of eligible patients who were not recruited into the trial, and their baseline characteristics, treatment preferences and intended treatment.

**Discussion:**

This article presents the protocol for a multi-centre randomised controlled trial. It gives extensive details of, and the basis for, the chosen methods, and describes the key measures taken to avoid bias and to ensure validity.

**Trial Registration:**

Current Controlled Trials ISRCTN50850043

## Background

### Rationale for the trial

Proximal humeral fractures account for approximately 4 to 5% of all fractures. Their incidence rapidly increases with age, and women are affected over twice as often as men. Similar to other primarily osteoporotic fractures, the incidence of these fractures is increasing. Palvanen et al. found a three fold increase over a 33 year period in the incidence of proximal humeral fractures resulting from low-energy trauma in people aged 60 and above [1].

A large prospective epidemiology study [2] found that around half of these fractures (51%) are displaced, when assessed according to the criteria of Neer's classification system [3]: one or more parts of the fractured bone are displaced by more than one centimetre, or angulated more than 45 degrees. Court-Brown et al [2] found that the largest groups of displaced fractures were 2 part surgical neck fractures (28% of the whole population), followed by 3 part greater tuberosity and surgical neck fractures (9%). Four part fractures without fracture dislocation were around 2% of the total. These figures are consistent with estimates from several members of the trial group.

Recent systematic reviews [4,5], one of which was updated in 2007 [4], have found a lack of evidence from randomised controlled trials (RCTs) to inform management decisions for proximal humeral fractures. In particular, there were only three completed RCTs comparing surgery with conservative treatment. All were small studies (numbers randomised: 30, 32, 40) with flawed methodology. Both reviews [4,5] concluded that it was unclear whether operative intervention, even for specific fracture types, would produce consistently better long-term outcomes.

It is also clear from the literature, confirmed by an informal survey of the treatment provided by several UK centres, that there is great variation in the treatment of these fractures, both in basic (the use of surgery) and specific (type of implants and surgical technique; non-surgical management [6] and rehabilitation packages) terms. Additionally, technology is changing all the time with various pressures towards early implementation.

The above findings point to a clear need to get reliable evidence to inform practice, and crucially to establish whether there is a role for operative intervention for the common types of acute displaced fractures of the proximal humerus. This is the focus of this trial.

### Trial aim

We aim to conduct a pragmatic multi-centre randomised controlled trial (RCT) to obtain good quality evidence of effectiveness and cost-effectiveness of surgical versus non-surgical treatment for the majority of displaced fractures of the proximal humerus in adults.

## Methods

### Overview

As indicated, we intend to undertake a pragmatic randomised clinical trial evaluating the effectiveness and cost-effectiveness of surgical intervention versus standard conservative therapy for the treatment of the majority of displaced (all involving the surgical neck) proximal humeral fractures in adults. This includes the systematic collection of reasons for non-inclusion of eligible patients who were not recruited into the trial, and their baseline characteristics, treatment preferences and intended treatment.

Underpinning our approach are two key issues:

• There is a general dearth of reliable evidence to inform on the use of surgery (definitive treatment) for patients with these fractures.

• There are known difficulties in recruitment and particularly patient (and surgeon) preferences. Based on experience from previous studies, some abandoned, in this field, we anticipated that a large proportion of eligible individuals are likely to refuse to be randomised because they (or their surgeons) will have a strong preference for one of the study interventions; generally conservative in the case of patients. On discussions with orthopaedic surgeons, lack of clinical equipoise, which is another important barrier to performing surgical RCTs [7], is anticipated to be less of an issue here. Because of these strong preferences, we consider that it is likely that patients recruited into any RCT will be a highly selected group, which may threaten the external validity of our study. Thus, collecting key data for all patients eligible for the RCT will allow us to set our randomised results within the context of the whole patient population and give some pointers to the applicability of the results of the study.

### Proposed use and interpretation of trial results

The trial aims to establish whether surgery yields superior results to non-surgical treatment. As detailed below, our protocol emphasizes standardised protocols and care pathways throughout, comparable and sufficient expertise of care providers and that the surgeon uses established techniques with which they are already familiar. Any questions over whether the use of other surgical methods, perhaps new methods, would give different results are countered by two considerations. Firstly, there is an absence of robust evidence to inform best surgical methods. Secondly, and arguably, the avoidance of 'learning curves' and the reliance on surgeon's competence is more representative of best surgical treatment.

### Brief details of the proposed practical arrangements for trial recruitment and treatment allocation

A detailed generic scheme of the recruitment process has been devised for adoption according to local circumstances in the participating centres. At radiological review by the surgeon or their nominated deputy, a trial eligibility form will be completed for any patient who meets the trial inclusion criteria. For ineligible patients, the surgeon is asked to indicate what treatment they would advise for the patient before the form is sent to the York Trials Unit (YTU). Those patients who the surgeon indicates as eligible for the trial will be invited to take part in the trial and the site-specific patient consent process is initiated. For non-consenting patients, this fact will be indicated on the Consent status form, where the surgeon or their deputy is asked to indicate their advised treatment, the patient's preferred treatment (if any), and the agreed treatment for the patient.

Once patients have given consent and their baseline form completed, the recruiting clinician will contact the York Trials Unit, either by telephone or via the internet, to access a secure randomisation service. This will ensure immediate and unbiased allocation of treatment.

**Figure 1:**
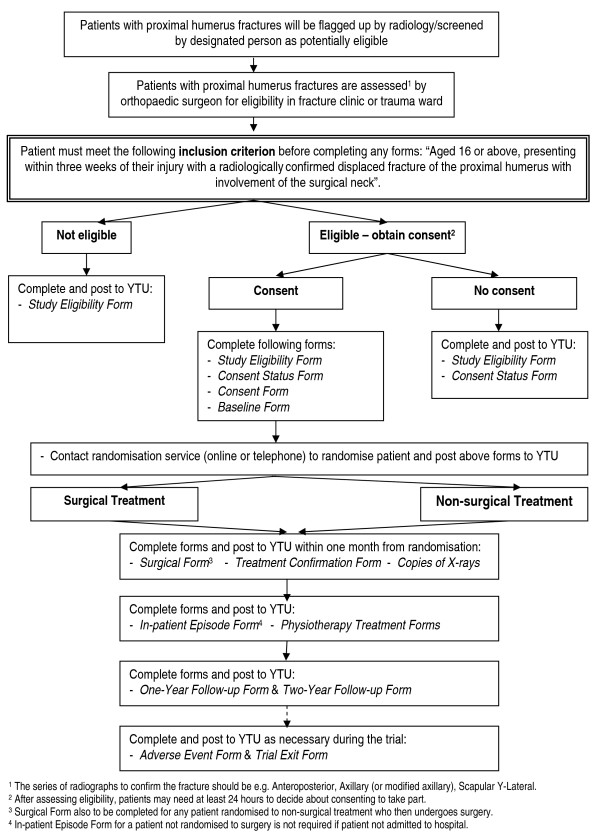
**Flowchart of patient recruitment and hospital data collection**.

A flowchart showing the patient recruitment and hospital data collection process is shown in Figure 1.

### Proposed methods for avoidance of bias and to ensure validity

Randomisation eliminates selection bias: there are, however, other forms of bias we guard against. We also take measures to ensure the external validity of trial results. We will undertake the following:

• Adherence to local guidelines for radiographic assessment will be actively promoted. If not stipulated already, we will encourage the use of the full shoulder trauma series [8]. Documentation including a power point presentation illustrating the full trauma series will be made available as part of the trial materials. A minimum of two radiographic views/projections is required for the assessment of study eligibility.

• At the end of the recruitment period, there will be scrutiny and categorization based on the Neer classification system, using pre-prepared forms, of the baseline radiographs of all randomised patients. This will be performed by an independent panel of musculoskeletal radiologists or orthopaedic surgeons who have experience with the Neer classification [3]. Copies of radiographs will be prepared beforehand to ensure they are anonymised. On an on-going basis during trial recruitment there will also be a review of the quality of the copies of the radiographs for each trial participant provided by trial centres. This is to ensure that at the end of trial recruitment the images are of sufficient quality for the independent panel to assess and classify the fractures. For the radiographs of the first five participants at each centre, independent assessment of the quality of images will be performed by three orthopaedic surgeons, one of whom will be the Chief Investigator. Assessment of the radiographs of subsequent participants of each centre will be performed by one orthopaedic surgeon (the Chief Investigator) who, if he has concerns, will ask the two other independent surgeons for their comments.

• Clear entry criteria, including checks at randomisation, will reduce inappropriate entry into the RCT.

• We will endeavour to provide a consistent approach to recruitment and obtaining informed consent by providing an unbiased account of the study to eligible participants using a specially produced information sheet. These materials have been produced in collaboration with service users.

• Concealment of treatment allocation prior to trial entry will be ensured by use of an independent telephone randomisation service, as provided by the York Trials Unit. After an initial, prespecified, period of randomisation, stratified by the presence or not of a tuberosity fracture and with a prespecified block size, randomisation will then be performed using a computer generated minimisation programme. The minimisation factors will be fractures involving either tuberosity and centre.

• We will emphasize good practice and standardised protocols and care pathways throughout, and comparable and sufficient expertise of care providers. Surgery for these types of fractures are usually carried out by consultants; this has been confirmed by an informal survey of the centres initially included in our study. We will attempt to minimise 'learning curve' issues for the surgical interventions by allowing the surgeon to use techniques with which they are familiar, but prohibiting the introduction of radically new or experimental methods during the recruitment period.

• We will encourage the prescription of comparable care, including rehabilitation programmes, such that any substantive departures from the norm would reflect the special requirements of a specific intervention. Consensus guidelines for rehabilitation for both groups have been prepared by rehabilitation specialists and were circulated for comment and input. These form part of the trial materials and we will request details of where the prescribed treatment differs substantively from the standardised protocols. (See Notes added in clarification below.)

Notes added for clarification

*1. Upon discussion, the proposed consensus guidelines for non-surgical management were considered inappropriate in the context of a pragmatic trial and the lack of evidence to inform practice. It was decided that the onus should be on the provision of good standard care and that our approach would be to indicate both verbally and in the site manual that we would anticipate initial care to comprise sling immobilisation for about 3 weeks or for as long as the treating clinician deemed necessary and active early rehabilitation. We considered that written information to advise patients during sling immobilisation was needed and should be provided to all eligible patients for the trial. We provide a generic document to be adopted by the hospital should a suitable document not already be available locally*.

*2. We stipulate that physiotherapy should be provided equally to both treatment groups. A consensus protocol giving basic treatment guidelines has been devised. Although, deviation from the protocol is allowed and expected, we stipulate that electrotherapy (except TENS) is not used, and point to an absence of evidence for these modalities as well as endorsement via a consultation process with specialist shoulder physiotherapists. We will promote the need to encourage home exercises, but decided not to provide generic information leaflets illustrating exercises for home use by patients. Instead we will check that physiotherapists either provide these already or access a standard web-based facilities to generate 'bespoke' exercise sheets*.

*3. We are prospectively collecting details of rehabilitation treatment which will also allow the detection of substantial differences from the physiotherapy protocol*.

• We shall follow the CONSORT guidelines for considering and reporting RCTs [9,10]. For instance, if eligible patients decline to be randomised, then this is refusal to consent (as per CONSORT); if surgeons choose not to randomise an eligible patient then this is a break in protocol (protocol violation) which must be recorded along with a reason.

• Intention-to-treat analyses will be undertaken as the primary analysis in the RCT.

• Active and systematic follow-up of all randomised participants at 3, 6, 12 and 24 months is planned. This will include pre-notification letters as well as the use of reminders after 2 and 4 weeks. For the 6, 12 and 24 month follow-ups, there will be an option for completion of an abridged questionnaire via telephone after 6 weeks. We will also include an unconditional incentive payment of €5 to maximize the 12 and 24 month follow-up.

• As far as possible, all participants will be followed-up for any unplanned events. Their hospital notes will include a reminder to notify of relevant subsequent treatment/events and they will be flagged for mortality. With the participant's permission, letters will be sent to their General Practitioners (GP) to inform of participation. Participant's permission will also be sought to allow us to ask their GP to provide the participant's contact details should there be problems contacting them directly.

• There will be independent data entry, processing and analysis. Aside from accrual and whole populations baseline statistics, interim results will not be made available to the trial investigators or associates in the participant centres.

#### Data collection on all potentially eligible patients

In addition to the systematic collection of basic baseline data for those eligible for the RCTs but who did not consent or where there was a protocol violation (reflecting lack of surgeon equipoise) to satisfy the requirements of CONSORT, we will collect data on patient-preferred and intended management. To complete the CONSORT flow diagram, we will collect the baseline data and reasons for ineligibility of ineligible adults presenting in the recruiting centres with the study fractures: see inclusion criteria.

#### Pilot study

The study was initially set up in Teesside (James Cook University Hospital, Middlesbrough) for training, and piloting materials and procedures.

### Ethics and impact on trial participants

MREC (Multicentre Research Ethics Committee) approval has been obtained from York Research Ethics Committee (reference number 08/H1311/12). Separate approval was also sought from local research ethics committee for each centre up until this became the responsibility of NHS R&D offices from 1^st ^April 2009.

In the context of the lack of robust evidence to determine the best treatment for patients with these fractures, the risks are not increased through trial and/or study participation. Measures, such as our emphasis on good practice and standardised protocols/care pathways throughout, taken by us are indeed likely to reduce risk and could bring additional benefits. We will emphasise the importance of surgeons performing operations with which they are familiar and undertake on a regular basis. We will also stress the importance of competence in conservative methods, principally rehabilitation. We will adhere to the good clinical research practice guidelines (MRC and Research Governance Framework). Our adoption of self completion questionnaires avoids the need for participants to specially return for clinical follow-up assessments.

The participant information sheet for the study, which has been compiled with involvement of service users, gives a balanced account of the possible benefits and known risks of the interventions under test. It states explicitly that quality of care will not be compromised if the participant decides to a) not enter the trial or b) withdraw their consent. Written informed consent will be obtained from all participants.

Each trial participant is identified using a unique identification number. Patient identifiable information will not be included in analytical datasets. All relevant trial documentation will be kept in a secure locality for a minimum of 20 years.

### Participants: planned inclusion/exclusion criteria

#### Inclusion criteria

Adults (aged 16 or above) presenting to the participating trauma centre within 3 weeks of their injury with a radiographically confirmed displaced fracture of the proximal humerus involving the surgical neck. This should include all 2 part surgical neck fractures; 3 part (including surgical neck) and 4 part fractures of proximal humerus (Neer Classification). It may also include displaced surgical neck fractures that do not meet the exact displacement criteria of the Neer classification (1 cm or/and 45° angulation of displaced parts) where this reflects an individual surgeon's equipoise (e.g., whether the surgical neck fracture should be treated surgically).

#### Exclusion criteria

• Associated dislocation of the injured shoulder joint

• Open fracture

• Mentally incompetent patient: unable to understand trial procedure or instructions for rehabilitation; significant mental impairment that would preclude compliance with rehabilitation and treatment advice

• Co-morbidities precluding surgery/anaesthesia

• A clear indication for surgery such as severe soft-tissue compromise requiring surgery/emergency treatment (nerve injury/dysfunction)

• Multiple injuries: same limb fractures; other upper limb fractures

• Pathological fractures (other than osteoporotic) & terminal illness

• Participant not resident in trauma-centre catchment area

#### Sample population

This will be all adults (aged 16 or above) presenting within 3 weeks of injury with fracture types listed in the inclusion criteria. Ineligible patients will be defined as those who are excluded for reasons given in the exclusion criteria. All those who meet the above criteria will be termed eligible patients. Some patients still may not be not included in the RCT, for instance due to lack of patient consent (patient has strong preference for specific treatment option or refuses randomisation) or because the surgeon considers one of the treatment options is strongly indicated for reasons other than above.

### Interventions

Each centre participating in this trial has to agree to forgo the introduction of radically novel and experimental interventions for these fractures during the recruitment period.

Central to the obtaining of reliable evidence is that good standard care, both surgical and non-surgical, is provided throughout the trial. Where possible, the decisions on the actual method of surgery, when allocated, and non-surgical treatment is left to the clinical judgement of the participating surgeon. Participating surgeons will be advised that they should, however, use surgical interventions and procedures with which they are familiar. This is to avoid learning curve problems. Similarly, physiotherapists are advised that they should use procedures with which they are familiar. The essential components of physiotherapy at each session will be recorded prospectively.

#### Surgery

**Figure 2:**
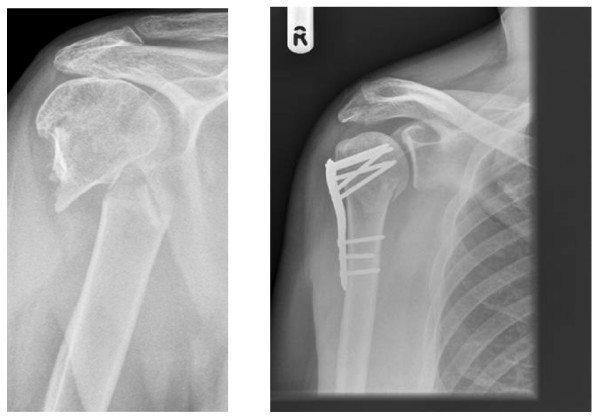
**Two-part fracture of the surgical neck with subsequent internal fixation**.

For displaced (2 part) surgical neck fractures: surgical interventions with which the surgeon is familiar. These are likely to be plate fixation or intramedullary nailing. For 3 part (including displaced surgical neck) or 4 part fractures: surgical interventions with which the surgeon is familiar. These are likely to include internal fixation such as nail, plate or other method which preserves the humeral head; or humeral head replacement (hemi-arthroplasty). See Figure 2 for an example of a two-part fracture of the surgical neck and subsequent internal fixation.

#### Peri-operative management

Peri-operative management including anaesthesia and analgesia, antibiotic and thromboembolism prophylaxis, and dressing policies will follow local guidelines.

#### Post-operative management

It is envisaged that similar rehabilitation packages, including mobilisation protocols, should be provided for all interventions. Specifically developed guidelines will be included in the materials for each centre.

#### Non-surgical intervention (the control group)

Brief recommendations for conservative treatment for trial participants will be included in the materials for each centre. Essentially, these advise that conservatively treated patients will be given sling immobilisation for about 3 weeks or for as long as the treating clinician deems necessary and active early rehabilitation. We will stress the need for competence in conservative methods, including rehabilitation.

#### Rehabilitation

As far as practical, centres are required to provide written advice on personal care during sling immobilisation to all eligible patients. A generic document has been devised that can be adopted by the centre if required. We will stress that similar access to physiotherapy should be provided for surgical and non-surgical participants. A basic treatment protocol for physiotherapy will be provided. This will emphasise that, while the protocol acts as a guide, variation in practice is accepted and anticipated. Electrotherapy other than TENS will be disallowed. We will promote strongly the need to encourage patients to perform home exercises and that they receive information sheets illustrating how to do the exercises.

### Outcome measures

The primary outcome measure is the Oxford Shoulder Score (OSS) assessed at 6, 12 and 24 months [11]. The Oxford Shoulder Score is a condition-specific questionnaire providing a total score based on the patient's subjective assessment of pain and activities of daily living (ADL) impairment. Consistent with recent developments, the range of available scores is 0 (worst) to 48 (best) [12]. The OSS contains 12 items, each with 5 categories of response. It has been shown to correlate well with both the professionally-endorsed Constant Score [13] and the SF36 assessment, and to be sensitive to clinical change at six months after surgical intervention [14]. It has been demonstrated to be consistent, reproducible and valid in a UK population [11]. This questionnaire will be administered by post for self-completion by the trial participant without need for an examination and thus avoids the requirement for follow-up visits to the clinic for assessment. To improve compliance, reminders will be sent and patients will be offered the option of completing the questionnaire via a telephone call. We will also send pre-notification letters and use unconditional incentives; both have been shown to be effective at improving response rates [15].

Secondary outcomes are:

• Surgical complications; including shoulder dislocation, failure of implant, proven wound infection (purulent discharge plus positive bacteriology or need for revision due to infection), septicaemia (clinical evidence of systemic infection plus positive blood cultures).

• Early medical complications, i.e. chest infection, confirmed MI or stroke (confirmed by senior clinician), treated DVT, treated pulmonary embolism and other serious event.

• Mortality, subsequent referral for operation or substantive treatment.

• The SF12 and Euroqol (EQ-5D) to collect general health status data (at 6, 12 and 24 months).

#### Data for economic evaluation

Prospective cost data on trial participants include costs incurred in hospital and subsequently. Thus, time spent in theatre and hospital consumables will be collected. Health utility data will also be obtained from the EQ-5D collected at 3, 6, 12 and 24 months [16]. Information for estimating NHS and societal costs will be collected from the trial participants at each follow-up.

We will collect data on the actual procedures performed, including anaesthesia, and interventions provided and the experience of operators/care providers (according to grade). We will collect these data for all trial participants.

### Data collection

We shall aim to make the trial processes as simple as possible in order to minimise the work entailed at the participating centres. As far as possible, we hope to achieve complete follow-up of all randomised patients.

#### Baseline data

Basic information including key baseline characteristics will be collected for all potentially eligible patients (i.e. those meeting the trial inclusion criteria) who are found not to be eligible.

Additional data on patient preferences, surgeon's advised treatment and the agreed treatment will be obtained for patients who do not consent to trial participation.

For consenting patients, we will collect data on ethnicity, education, employment, previous fractures, shoulder dominance, injury mechanism, smoking, diabetes, treatment preference, current health status (EQ-5D), GP name and surgery and the patient's contact details.

#### Description of treatment

##### Surgical methods

Brief details of the actual surgery and procedures used will be recorded by the surgeon, or assigned deputy, following the operation. Also prescribed rehabilitation.

##### Non-surgical methods

Brief details of the prescribed non-surgical treatment will be recorded by surgeon, or assigned deputy. Also prescribed rehabilitation.

#### Collection of hospital outcome data - before hospital discharge

Centres will be required to complete data forms detailing:

• Clinical outcomes including surgical complications and early medical complications

• Resource use: the data on hospital costs will be collected using a cost proforma designed for the trial.

• Substantive deviations from prescribed treatment and rehabilitation

• Patient destination after hospital discharge

#### Long term follow-up

One and two year follow-up forms from centres

Forms to notify mortality or subsequent surgery for completion and return at any time will be made available for the completion by centre staff. Forms for completion will be sent at 1 and 2 year follow-up - it is likely that all the data for these can be gleaned from the hospital records for these patients.

#### Follow-up patient questionnaires: 3, 6, 12 and 24 months post trial recruitment

A short questionnaire including the EQ-5D and brief questions on the number of consultations of NHS care providers (GPs, physiotherapists, district nurses etc), hospitals attendances, use of private healthcare and days lost from work or other normal activities will be sent together with a covering letter by the YTU to all participants at three months. Reply paid envelopes will be included. Reminders will be sent after 2 and 4 weeks

Full questionnaires, together with covering letters and reply paid envelopes, will be sent by the YTU to all participants at six, 12 and 24 months after recruitment. These include the Oxford Shoulder Score, EQ-5D and SF12, all of which are self completion questionnaires. As at 3 months, brief questions on the number of consultations of NHS care providers (GPs, physiotherapists, district nurses etc), hospitals attendances, use of private healthcare and days lost from work or other normal activities will also be requested. Reminders will be sent after 2 and 4 weeks and options for completion of the questionnaires via telephone after 6 weeks. If completed over the telephone, only the Oxford Shoulder Score, EQ-5D and information on hospital readmissions will be requested. An unconditional incentive payment of £5 will be sent for the 12 and 24 month follow-ups [15].

### Radiographs

Copies of all baseline radiographs for all randomised patients will be requested for independent and blinded assessment at the end of study recruitment. Radiographs will also be reviewed by local experts on an on-going basis during trial recruitment to ensure the images are of sufficient quality for scrutiny and classification based on the Neer classification system by an independent panel of experts at the end of recruitment.

### Sample size

The primary outcome for the trial is differences in patients' subjective assessments of pain and activities of daily living (ADL) as measured by the Oxford Shoulder Score (OSS). For surgery to be worthwhile, it needs to demonstrate greater improvements in patient's subjective assessments of pain and ADL than those for conservative treatment to justify both its increased costs and the exposure to the hazards of surgery. In an observational study conducted by one of us (AR) it was found that those patients who had surgery had a 5 point differential improvement in the OSS compared with those patients treated conservatively. Given a standard deviation of 12 this equates to an effect size of 0.42. We propose, therefore, to design the study to observe an effect size of 0.4 at 80% power using 5% significance level, which would require approximately 200 participants. After allowing for drop-outs of 20%, we propose to recruit and randomise 250 patients (125 surgery and 125 controls). Our estimate of 20% loss from the RCT is purposefully pessimistic for sample size calculations.

#### Recruitment rate

We anticipate that recruitment for this trial will be potentially challenging. Therefore, we have set very conservative recruitment targets. Our recruitment period is 18 months. We aim to recruit between 18-20 centres. Each centre will be expected to recruit only one participant per month, though encouraged to aim higher than this. We estimate across 18 centres there will be 6066 patients (6000 is used as a working figure here) with a proximal humeral fracture over the 18 months of recruitment. Of these, 2391 (thus, 2400 is used as a working figure below) will have the fracture types suitable for inclusion into the RCT. To achieve our sample size we need to recruit only 11% of these patients.

#### Loss to follow-up

We anticipate that the main reason for loss to follow-up will be mortality. We will follow up patients assiduously using postal questionnaires and in the event of non-response we will contact their GP to ascertain whether it is appropriate to contact the patient and, if so, their address.

### Statistical analysis

All of the analyses will use the intention-to-treat principle. Consequently, any patients who cross over from either study arm will be analysed as per their randomisation status. The primary outcome is the Oxford Shoulder Score (OSS). The difference between the two treatment groups will be compared over all follow-up assessments (i.e. 6, 12 and 24 months) using a repeated measures model. The model will include terms for treatment, follow-up time, and also adjust for type of fracture, age and gender (as older people and women are more likely to sustain these fractures). Because participants are clustered by surgical centre there is a theoretical possibility that there may be a 'surgeon' effect. We will therefore repeat the primary analysis using appropriate statistical techniques (robust standard errors) to account for the clustering of patients within surgeon. The anonymity of individual surgeons and centres will be preserved for all analyses and there will be no presentation or comparisons of the treatment results from individual centres or surgeons. Subgroup analyses based on the Neer classification system are planned to assess the effectiveness of treatment for the different fracture groups (2 part surgical neck; 3 part including surgical neck and 4 part fractures; fractures not meeting the Neer classification displacement criteria). The secondary outcomes will be summarised for each treatment group.

#### Frequency of analyses

We anticipate that there will be a single analysis at the end of the study. However, decisions about the need for interim analysis will be taken by the chair of the independent Trial Steering Committee (TSC) in conjunction with the chair of the Data Monitoring and Ethics Committee (DMEC).

#### Assessment of study recruitment and applicability

We will report the numbers of and reasons for ineligible adults (aged 16 or above) with the study fractures, we will also report the numbers of and reasons for the non-inclusion of potentially eligible patients. We will compare the baseline characteristics, patient preferences with those of randomised patients.

### Economic evaluation

An economic analysis will be taken from the perspective of the UK National Health Service and Social Services. The horizon for the baseline analysis will be two years. However, we will model any potential benefits forward to 5 years and an average lifetime in a sensitivity analysis.

Health benefits for the economic analysis will be measured in terms of quality adjusted life years (QALYs). Health utility values for individuals with displaced proximal humeral fractures will be estimated using the Euroqol (EQ-5D) questionnaire. QALYs will be calculated for each patient using the area under the curve defined by her/his EQ-5D scores over the two-year follow-up period and adjusted by the Kaplan Meier estimates of patients' survival over the same period of time. Given the horizon for the analysis is longer than a year a discount rate of 1.5% will be applied to health benefits [17].

Resource use and clinical data will be collected for all trial participants. Information regarding total volume of resources used in the treatment (conservative/surgical) and rehabilitation procedures will be recorded for each patient. Unit cost will then be applied to estimate the total cost per patient. To account for the censored nature of cost data, the Lin method will be used to estimate the mean average total cost per treatment arm [18]. Non parametric bootstrapping techniques will be used to estimate 95% confidence intervals for the mean differential cost between conservative and surgical treatment [19]. Total cost will be discounted using a 6% annual discount rate [17].

Health benefits and mean average total costs associated with each of the trial arms will be combined in a cost-utility analysis, incremental costs per quality ratios will be computed comparing the conservative and surgical treatment interventions for adult patients with a displaced proximal humeral fracture. Multilevel modelling will be used to explore potential variations in treatment effect and costs between health professionals [20].

### Trial timeline

After a six-month period for trial preparation, obtaining ethics approval, and establishing and piloting of all trial materials and processes, the official start date for full-trial recruitment was set at 1^st ^October 2008. The planned recruitment period is 18 months, and completion of follow-up 24 months after that. Study completion, which includes submission of the draft trial report to the funders for publication in the HTA Journal Series, is scheduled for 1^st ^October 2012.

### Dissemination of trial findings

We shall disseminate our findings through relevant local, national and international conferences and peer-reviewed publications. Reflecting the collaborative basis of this research, all active contributors will be named and credited in the main report.

### Trial management

The day to day management of the project is the responsibility of the Trial Management Group:

• Clinical co-ordination: Amar Rangan (Chief Investigator)

• Trial management: Stephen Brealey (Trial Manager, University of York) and Laura Dennis (Trial Co-ordinator, Teesside University)

• Methodological support: Helen Handoll (Teesside University), David Torgerson (University of York)

The trial co-ordinating centre is York Trials Unit. Specifically assigned to the ProFHER trial are a Statistician (Mrs Gill Worthy, replaced by Dr Catherine Hewitt (April 2009)), a Health Economist (Dr Jo Dumville, replaced by Miss Ling-Hsiang Chuang (May 2009)), Data Managers (Mr Ben Cross and Mrs Valerie Wadsworth), and a Trial Secretary (Mrs Sarah Gardner).

## Discussion

This article describes version 7.0 (19/05/09) of the protocol for the ProFHER trial. Various adjustments, all approved by ethics, have been made to the original protocol approved by ethics. Many changes were clarifications in wording. A few others were in response to feedback, such as from the independent members of the Trial Steering Committee, and to overcome practical barriers in trial recruitment. An example of changes is presented in *Notes added for clarification *in the Methods section. All changes, which have been fully documented, are likely to improve the prospects of the trial successfully meeting its aims.

## Competing interests

The authors declare that they have no competing interests.

## Authors' contributions

All of the authors contributed to the design and development of the trial protocol. HH and SB were responsible for writing this manuscript. All authors read and approved the final manuscript.

## Pre-publication history

The pre-publication history for this paper can be accessed here:

http://www.biomedcentral.com/1471-2474/10/140/prepub
